# Male graduates transitioning into the workplace: managing stress through the sense of coherence components

**DOI:** 10.3389/fpsyg.2023.1053173

**Published:** 2023-05-05

**Authors:** Kiana Fayard, Claude-Hélène Mayer

**Affiliations:** Department of Industrial Psychology and People Management, University of Johannesburg, Johannesburg, South Africa

**Keywords:** salutogenesis, sense of coherence, stress, young male, transition from university to the workplace

## Abstract

Male graduates are faced with many challenges when transitioning into work life after graduation. This transition from university to the workplace is one of the most important developmental stages in a young adult's life. It has an important impact on their careers and causes increased stress levels. Often, young men are suffering from mental health challenges and feel as though they cannot seek the appropriate help. Thus, it is necessary to determine how young male graduates cope with the changes experienced in this period, especially relating to their sense of coherence and salutogenesis. The aim of the study is to investigate the transition from university to the workplace and to understand their stress and well-being experiences in terms of activating the three sense of coherence components for coping. A qualitative approach is employed through the use of semi-structured interviews with 10 male South African university graduates. A content analysis technique was used to analyse the qualitative data. The findings indicate that most of the young male graduates understand the transition from university to the workplace and the challenges that come with it (comprehensibility). They also have the necessary personal resources to cope with the stress (manageability) while experiencing this life phase as meaningful (meaningfulness). To understand the transition into the workforce was the most important aspect to stay health during the transition. However, male graduates mainly coped by applying their personal coping strategies and mechanisms and thereby managed their transition mainly by themselves, not based on organizational structures or integrative processes. Meaning applied to the transitional process mainly derived from their personal concepts of creating a meaningful life, not as such from the meaning applied to the work or position they held. The findings provide insights which can be used by higher education institutions to prepare graduates for the transition into the workforce and for organizations to develop programmes for graduates to improve their transition into the organization.

## 1. Introduction

Young adulthood is a critical developmental stage which has profound and long-lasting implications and impacts on people's lives. These implications and impacts contribute to the people's employment and career paths, their economic security and to their physical, psychological, and emotional well-being (Mayer, 2014). The world has changed drastically in recent decades; there are increasing demands on young adults, with less latitude to fail (Stroud et al., 2015).

The increased global competition for jobs, coupled with the structural changes within the labor force, work has become less stable for employees entering into the labor force (Aronson et al., 2015). Apart from these changes, graduates enter into a workplace that is characterized by economic instability and challenges, resulting in them being less likely to find a suitable job that matches their knowledge and skills (Monteiro and Almeida, 2015). These insecurities in work contexts cause stress, which can be defined Ivancevich and Matteson (as cited in Oosthuizen and van Lill, 2008, p. 64) as the

“adaptive response, mediated by individual differences and psychological processes, that is a consequence of any external (environmental) action, situation, or event that places excessive psychological and physical demands on a person.”

Stressors impact on employees functioning in their day-to-day lives, often leading to a change in their normal functioning patterns (Oosthuizen and van Lill, 2008). When employees experience stress, they may have a positive response that encourages and motivates them in their work, or they may have an adverse reaction which may cause distress (Oosthuizen and van Lill, 2008). Therefore, stress occurs when the magnitude of the stressor that is experienced exceeds that individual's capacity to cope (Rothmann and Malan, 2006; De Simone, 2014; Braun-Lewensohn and Mayer, 2020).

The transition from university to the workplace is a crucial developmental stage for graduates of male and female gender, as the literature shows (Maxwell and Broadbridge, 2014; Papafilippou and Bentley, 2017; Melendro et al., 2020). During this transition, the graduates will put to use the skills and knowledge they have gained in the context of the work environment. However, at times when graduates start working, they are challenged by the realities of the working world, more specifically the work environment, job security and non-technical tasks (Baytiyeh and Naja, 2012). Additionally, there are specific skills and knowledge that may be required in an organization for high productivity and performance. Therefore, graduates not only face the challenges associated with their skills gap and knowledge, but they also have to manage the possible disappointment resulting from unmet expectations (Baytiyeh and Naja, 2012).

There is a lack of research conducted of young male graduates transitioning from university to the workplace in general (Cheng et al., 2016) and with regard to salutogenesis (Mayer, 2014). Therefore, it is necessary to carry out more research to determine how the graduates cope with these specific changes. To manage the change and meet the challenges of entering into the workplace, one needs to have a strong sense of coherence to comprehend and manage it in a resourceful manner. Moreover, it is important to determine what makes the young male graduates work meaningful, to give them the motivation to carry on in their chosen careers.

Based on a comprehensive literature review the main research question is: How do young male graduates, specifically working within the private sector in South Africa, experience the transition from university to the workplace in relation to their SOC levels? The SOC factors of comprehensibility, manageability and meaningfulness can help to determine the aspects in an individual's life that give them the means to cope with stress they experience within the workplace. By making use of the salutogenesis and SOC framework, researchers will be able to understand the experience that young male graduates have had while transitioning from university to the workplace, especially during COVID-19. Moreover, it will determine how the graduates have been able to manage the demands they have experienced during this transition period. Finally, it will determine what makes the transition meaningful for them while coping with the demands.

## 2. The transition from university to the workplace

The transition that young adults go through from university to the workplace is a critical developmental period (Baytiyeh and Naja, 2012; Haase and Heckhausen, 2012; Melendro et al., 2020). The success of this transition poses essential long-term effects on graduates mental and physical health, their personality development, social relationships, and future career success (Haase and Heckhausen, 2012; Tuononen et al., 2019). These new changes in the graduate's life may change their assumptions about themselves and the world, resulting in them needing to change their behavior and relationships (Griffith et al., 2013; McBeth et al., 2017).

Currently, there is an increase in the global competition for jobs and the structural changes within the labor force, making work unstable for graduates entering the labor force (Aronson et al., 2015; Monteiro and Almeida, 2015). This is specifically the case in South Africa: At the end of is the first quarter in 2021, Statistics SA published that the unemployment rate among the youth (ages 15–34 years) had reached 46.3%; of that 2.1% were unemployed graduates, and 7.5% had other tertiary qualifications as their highest level of education (Statistics South Africa, 2021). Additionally, some graduates may enter into a temporary contract and are left facing difficulty in finding permanent employment (Tuononen et al., 2019).

Aronson et al. (2015) show that adapting to the workplace schedule is challenging as some graduates found it challenging to get used to working a 40-h workweek, not having the same amount of free time and work being less “spontaneous” or “enjoyable” then university life (Aronson et al., 2015). Thus, this can also contribute to graduates' inaccurate perceptions of the working world and work-life (Rothmann and Malan, 2006; Wendlandt and Rochlen, 2008).

Graduates also face the challenge of lacking specific skills and experience when entering the workplace (Wendlandt and Rochlen, 2008). Although universities teach graduates essential skills and knowledge regarding the subject they are studying, skills such as communication may be lacking (Baytiyeh and Naja, 2012). Graduates may come across as being underprepared to fit into the company's culture, they may not understand the politics in the corporate world, function within the structures of power and rewards, may have difficulty building effective working relationships, be accepted by their team members, and earn respect and creditability from their colleagues (Baytiyeh and Naja, 2012). Thus, these challenges may impact the graduate's self-confidence in their skills, abilities, and competence in their position, causing the graduates to experience extreme amounts of stress within their first few years of employment (Baytiyeh and Naja, 2012).

## 3. Stress in young men and the impact of COVID-19 on the workplace

Men and women are exposed to different work environments and different types of job demands and tension, even when working within the same company, position, or sector (Rivera-Torres et al., 2013). When considering graduates' experiences of this perceived stress, studies mainly focus on women only, with a few studies focusing on men only.

Stress is considered to be critical determinate of African American men's health which ultimately, directly and indirectly, contributes to their high rates of unhealthy behaviors, chronic disease diagnosis, and premature mortality among men (Griffith et al., 2013). Within the South African context, past research has focused on female Colored women and stress, and only very few studies explore men's stress and coping mechanisms in the workplace (Watson et al., 2011). However, research on young male graduates from different cultural background entering the South African workplace is extremely limited and therefore strongly needed.

Evidence has shown that there is a connection between work-related stress and various physical and mental health problems (Bowen et al., 2014). This stress can result in either physical (e.g., sleep, headaches, and gastrointestinal upsets), emotional (e.g., anxiety and depression), intellectual (e.g., decreased concentration and lack of motivation) and behavioral (e.g., substance abuse, absenteeism, and poor motivation) disturbances (Bowen et al., 2014; Stroud et al., 2015). The transition from university to the workplace can be a very stressful event. However, when the stress is too overwhelming for men, they may not seek the necessary professional help to cope with it (Oliffe and Han, 2013; Lynch et al., 2016). Additionally, young men may experience a stigma, as well as discomfort, embarrassment, fear and shame when required to ask for help (Lynch et al., 2016). This may play into the idea that men need to be “macho” and “man up” and that seeking professional help for feelings of stress may mean that they are seen as lesser men (Lynch et al., 2016).

This COVID-19 pandemic has caused even more significant disruptions in people's everyday functioning, global economic recessions that have come with enormous financial volatility and caused extreme stress (Ruiz-Frutos et al., 2021; Yu et al., 2021). To adapt to these changes, organizations and employees now need telecommute, forced to work from home in order to practice social distancing and limit the spread of COVID-19 (Palumbo, 2020). Oakman et al. (2020, p. 2) defined telecommuting/telework as making “use of information and communications technologies (ICTs) including smartphones, tablets, laptops, or desktop computers for work that is performed outside of the employer's premises”. These changes add to the widespread and uncontrollable stress, when compared to the stress experienced in pre-pandemic everyday life (Yu et al., 2021).

Evidence suggests that starting a new position is often characterized as a time that is uncertain and potentially can be a very stressful experience, even more during the implications caused by the COVID-19 pandemic (Ployhart et al., 2021). Moreover, the pandemic has led to an increase in economic and health stressors that people face, which can lead to employees feeling depleted in their personal and social psychological resources to cope (Ployhart et al., 2021). Further, job enterers during the pandemic experienced the mixing of the physical and organizational boundaries between home and work as stressful and difficult, also due to the longer work hours, lack of boundaries between work and home life and the limited social support of the teams and organizations (Oakman et al., 2020).

## 4. The salutogenic approach

With the paradigm shift from a negative toward a positive health paradigm, theories have moved from a pathogenic approach that focuses on the harm-causing potential in the work environment (Roskams and Haynes, 2020) and ways that stressful life events pose themselves on an individual which causes a variety of negative health outcomes (Pallant and Lae, 2002; Bauer et al., 2020) toward a salutogenic approaches (Antonovsky, 1979, 1987a,b).

Salutogenesis focuses on enabling employees to increase their control over, and enhance, their overall health and well-being. Thus, organizations must create appropriate conditions that will assist health and well-being behaviors. Furthermore, the salutogenic approach focuses on the factors that encourage health and well-being instead lowering them (Ruohomäki et al., 2015). This theoretical approach was used in this study to explore the resources young male graduates use to manage their work start during COVID-19.

This functioning within an individual is characterized on three different levels. Firstly, on a cognitive level, individuals will perceive environmental stimuli positively and constructively and use that information to make effective decisions. Second, an individual will be maturely committed to their life, self-awareness, confidence, and self-fulfillment on an affective level. Finally, on a motivational level, individuals will be able to perceive stimuli within their environment as a personal challenge and channel their energy to cope with stressors, solve problems and achieve results (Oosthuizen and van Lill, 2008).

Antonovsky (1979) explained that an individual's state of health should not be focused on only the disease, but we should instead look at it as a continuum between ease and dis-ease (Nilsson et al., 2012). Therefore, it is a movement along this continuum that results in competing forces that influence an individual's state of health or well-being (Roskams and Haynes, 2020). An individual will face unavoidable everyday stressors and hardships that contain the pathogenic potential to drive them toward ill health and disease. However, individuals will also be able to draw on the generalized resistance resources (GRR) that will enable them to successfully cope with or avoid these stressors and prevent tension from being translated into stress (Roskams and Haynes, 2020). It then has the potential to promote more positive health outcomes for the individual (Roskams and Haynes, 2020). Furthermore, an individual's position on this continuum will be determined by the interaction of the environmental threats (stressors), their degree of resistance (GRR), and the strength of their sense of coherence (SOC) (Mayer, 2011).

The SOC is the feeling of confidence that an individual has when faced with different life events, that they have the resources to face and cope with the demands of these events, and experiencing them as meaningful and worthy of engagement (Pallant and Lae, 2002). According to Antonovsky (1993), when confronted with a stressor, an individual with a strong SOC will:

- Know and trust that the challenge that they are facing is understood (comprehensibility).
- Know and trust that the resources that they need are available (manageability).
- Wish to be motivated to cope (meaningfulness).

These three main SOC components describe how an individual experiences and values a stressor concerning their capacity in terms of their understanding (comprehensibility), their ability to use their resources (manageability), and their personal commitmen to (meaningfulness) (Nilsson et al., 2012; Barnard, 2013; Bauer et al., 2020).

## 5. Research methodology

This study adopted a phenomenological (hermeneutic) design, combining phenomenological and hermeneutic views (Mayer, 2011). The premise of phenomenology is studying the lived experiences or real-life world, lived by the person and not by the world or reality that is separate from the person (Laverty, 2003). Thus, it takes an interest in understanding a phenomenon directly from the viewpoint of the participants involved in it (Venter et al., 2017).

This study adopts purposive sampling and snowball sampling strategies to determine the sample. The purposive sampling technique is used for “identification and selection of information-rich cases” (Palinkas et al., 2015, p. 534). Therefore, using this sampling technique aims to expand the depth and not the breadth of our understanding of the participants' experience (Campbell et al., 2020), allowing researchers to make the most of the limited resources available (Palinkas et al., 2015).

The sample consists of ten participants (P) who are young male graduates working within the Private Sector in South Africa in a variety of industries from engineering, law, fast moving consumer goods and information technology. All participants have obtained a South African qualified University degree, enter the South African workforce, are over 18 years old and speak English fluently.

### 5.1. Data collection and analysis

Data were collected through semi-structures interviews and aimed to explore participants' experience, emotions, processes, and practices in relation to their experience transitioning from university to the workplace (Venter et al., 2017). It also allowed for probing questions to be asked if extra elaboration was needed (Venter et al., 2017). Thus, the interviews consisted of 13 predetermined questions covering the SOC components of comprehensibility, manageability, and meaningfulness, while allowing the interviewer the opportunity to explore other ideas that may have arisen within the interview. the interview questions were developed based on an in-depth investigation of the literature and the constructs being measured within the Life Orientation Questionnaires of the Sense of Coherence (13- and 29-item scale) (Antonovsky, 1979, 1987a). Example questions adopted during the interview are:

- “How do you usually navigate your way through a difficult situation/challenge in your job?”—relates to comprehensibility
- “What makes your job meaningful?”—relates to meaningfulness
- “How do you manage your job?”—relates to manageability

The interviews were conducted virtually, making use of applications such as Zoom and Microsoft Teams. During the interview, the researcher introduced herself, explained the research study and built a trustful relationship with the interviewees, as far as this was possible within the given time frame (max. of 2-h). Finally, the researcher spent adequate time collecting data in the case-study sites to ensure that there was a full understanding of the constructs being researched. The lack of any new emerging data showed that data saturation had been achieved and the interview process stopped after conduct of 10 interviews (Houghton et al., 2013). Interviews were recorded and transcribed verbatim.

For the qualitative data set, the study employed content analysis defined as a “research method that provides a systematic and objective means to make valid inferences from verbal, visual, or written data in order to describe and quantify specific phenomena.” (Downe-Wamboldt, 1992). It focuses on exploring the meanings, intentions, consequences, and context of the data (Downe-Wamboldt, 1992), by making use of existing theories, research and literature, key themes, concepts, or variables which have been identified as the initial coding categories (Hsieh and Shannon, 2005). To analysis the gathered data, the study followed the 5-step process outlined by Terre Blanche et al. (2006): Step 1: Familiarization and immersion. Step 2: Inducing themes. Step 3: Coding. Step 4: Elaboration. Step 5: Interpretation and checking. Codes that were applied to the SOC-components were counted to present frequencies which show the emphasis of the three components. The use of frequencies in content analysis regarding SOC components has been used in previous research (e.g. Mayer, 2011) and was adopted here.

### 5.2. Quality criteria and ethical considerations

Credibility within this study was achieved through thick descriptions, crystallization of data, providing evidence that could be used for a wide range of stakeholders (organizations and young male graduates) and engaging in member reflections with the participants (Tracey and Hinrichs, 2017). This study ensured transferability by discovering meaningful findings through thick descriptions, explaining the sampling strategy (Walsh and Downe, 2006), research methods, the examples of the raw data used (Houghton et al., 2013) and describing the findings in relation to the existing literature (Walsh and Downe, 2006). The study ensured that dependability was reached by answering the research question based on the findings it produced by using appropriate methods that fit its paradigmatic and research stances (Tracey and Hinrichs, 2017). To show that confirmability can be achieved, the study outlined details with regards to the general methodological procedures, such as data collection, transcription, analysis and interpretation of data (Mayer, 2011).

Ethical consideration were applied on the proper treatment and protection of the participants throughout the research (Lefkowitz, 2017), including: gaining ethical clearance to gather the data, creating informed consent given to the participants, respect the right of the participant to withdraw from the data collection process, protection of confidentiality, the respect regarding participants, data, and transparency. Ethical approval of this study was provided by the University of Johannesburg, Johannesburg, South Africa.

## 6. Findings and discussion

The findings will be presented and discussed with regard to the three SOC components.

### 6.1. Comprehensibility

The comprehensibility component refers to the understanding of the world of the participants.

#### 6.1.1. The overall transition

In terms of comprehensibility, “The overall transition” (Table 1) is a major issue for graduates who have varying experiences, such as simultaneously working and studying and the COVID-19 pandemic. Four participants felt as though their transition was relatively easy:

“*For me, that was different it was learn a lot, try a lot, a lot of uncertainty in the sense of, also, you know you now not just dealing with people at your university but different universities from around the country, different degrees, different backgrounds. So, I think it was relatively easy”* (P1^1^, White, Bachelor of Commerce (Honors) in Marketing Management, working in the FMCG industry).

**Table 1:** Transition toward the workplace.

**Frequency**	**Code**	**Participant**	**Category**	**Frequency**
4	Easy transition	P1, P2, P3, P7	The overall transition from university to the workplace	10
2	Smooth transition	P5, P6		
4	Difficult or challenging transition	P4, P6, P8, P9		
3	Working and studying	P3, P7, P10	Working and studying	3
5	Overtime/long hours	P3, P4, P6, P8, P10	Change from university to the workplace	21
4	Confidence	P4, P5, P6, P10		
5	Uncertainty	P1, P4, P5, P9, P10		
7	Experience	P2, P3, P5, P6, P8, P9, P10		
5	Understanding the complexities of the workplace	P1, P2, P5, P7, P9	Understanding the workplace	8
3	Understanding the work environment	P4, P6, P9		

Others highlighted the impact of Covid-19 (P2, White, Bachelor of Commerce in Informatics and Economics, working in the Information Technology Industry):

“*…probably not like everyone else's. Because when I started, I only worked for two months, and then COVID hit. So, I started work, working from home, and it was very relaxing and never had to go in, and in my line of work, it was very easy to work from home. And it also eliminated a lot of stress.”*

Other participants described their transition as smooth and the acceptance of not knowing anything initially gave them the ease to transition from university to the workplace. However, four participants described their transition to be full of challenges and difficulties (P8, Black, Bachelor of Law Degree, working in the legal industry):

“*It was difficult. Still is difficult. I say because I think the biggest adjustment is just time wise.”*

One participant described entering the workplace from university as a culture shock. Another participant stated that the biggest challenge was adjusting to the time limits and constraints. He felt as though the challenge for him came in the form of a knowledge and skill gap, describing that

“In [his] first year, it was it was completely different. [he] felt like [he] had no idea what [he] was doing the majority of the time.” (P9, White, Bachelor of Arts (Law) and Bachelor of Law Degree, working in the legal industry).

Three participants all described their transitions as relatively easy or smooth, because they had started working while they were still studying. This helped them understand the workplace and know what to expect in their overall transition:

“*So, what happened was I was I started working while I was still studying. So, I feel like the going over from studying to working was a lot easier for me, because I did it little by little”* (P7, White, Master's degree in marketing, working in the Wine industry).

The participants all stated that their transitions from university to the workplace were met by many changes to their lives. Five participants stated that their work consumed a lot of their time outside of work:

“*It doesn't remove the stress, anxiety of the deadline being looming, and it ends up with me having to work over weekends to compensate for it.”* (P4, Black, Bachelor of Engineering in Mechanical Engineering and a Bachelor of Engineering (Honors) in Industrial Engineering and working in the Consulting Industry).

Four participants felt that it was difficult to adjust to the working hours and that the workload was more than what they had experienced in university.

“*I have a problem with, with, with like the nine to five not in a, I don't know how to put it I just in that in that, like, in that sphere, the fact that this is now my life compared to what I had gotten used to as a master's degrees student, it's not that nice (laugh).”* (P6, Black, Master of Science Degree in Environmental Science and Remote Sensing working in the ICT industry).

Some participants stated that they started to question their abilities, skills and knowledge they had gained at university, which did not align with the workplace demands. From these statements, we can deduce that the participants felt their confidence was lowered when they were tasked with a project or work. However, one participant used his confidence to have a more positive experience transitioning from university to the workplace.

Further, uncertainty was a common theme throughout the interviews. Five participants stated that they felt a sense of uncertainty in their new positions. This uncertainty stemmed from what exactly their job responsibilities were, what was expected from them and the standard of work that was required from them. Another five participants stated that the main source of their uncertainty during this time was the lack of clear guidelines on what was exactly expected of them in their work. Seven participants expressed that they lacked a certain level of experience which played a role in their transition from university to the workplace. The participants highlighted that when they started their positions, they did not have the required experience that was needed for the level or type of work that they were tasked with. Finally, two participants explained that they need to understand the workplace and its complexities, as well as the work environment which they managed by trial and error or asking others for help (see Table 1).

#### 6.1.2. Relationships

All of the participants described their relationships with their co-workers, with this theme broken down into five categories (Table 2).

**Table 2:** Relationships at work.

**Frequency**	**Code**	**Participant**	**Category**	**Frequency**
8	Good relationships	P1, P2, P3, P4, P5, P6, P8, P10	Relationship with co-workers	18
3	Professional relationship	P3, P7, P10		
5	Supportive relationship	P1, P4, P6, P7, P8		
1	Relationship outside of team	P6	Relationship with colleagues outside of the team	1
1	Rotation of teammates	P8	Rotation of teams	1

Eight participants stated that they had good working relations with their co-workers. However, this positive relationship were found to only extend within the participants direct team and not to other colleagues working for other teams or departments.

“*I think in, in, the team that I work with, there was a good relationship a good report, we were happy team”* (P1, White, Bachelor of Commerce (Honors) in Marketing Management, working in the FMCG industry).

Three participants stated that their relationships with their co-workers is one that was strictly professional in nature and that they do not socialize outside of work.

“*It's strictly professional. Apart from those ones, where I kind of have beers with every now and then every weekend. But with everyone else is just strictly collegial, strictly professional”* (P10, Black, Bachelor of Law Degree, working in the legal industry).

Five participants stated that they had supportive relationships with their co-workers. This means that they supported each other in their work when they did not understand what needs to be done, or if other co-workers needed further assistance in cases such as having COVID-19.

“*We chat a lot, we support each other, and we see it when you know somebody's struggling with something I think it's really been tested a lot now with COVID”* (P1, White, Bachelor of Commerce (Honors) in Marketing Management, working in the FMCG industry).

One participant stated that he felt as though the relationship that he had with colleagues who did not fall within his direct team was one that came with favors. Furthermore, one participant stated that when it is time to rotate teams in the workplace, the time it took to adjust and build the relationships with the new team was not a positive one right away.

#### 6.1.3. Transition through learning

Five participants stated an eagerness to learn in the early years of their career (Table 3). The learning encompasses the environment that the participants are in and the potential to learn something new about their line of work.

“*I think there's a lot of environments or a lot of potential that I can do to learn”* (P1, White, Bachelor of Commerce (Honors) in Marketing Management, working in the FMCG industry).

**Table 3:** Learning.

**Frequency**	**Code**	**Participant**	**Category**	**Frequency**
5	Learning	P1, P3, P5, P7, P9	Learning	5

#### 6.1.4. Transition through COVID-19

The COVID-19 pandemic has played a crucial role in the experience that the participants have had while transitioning from university to the workplace.

Six emphasized that they felt robbed or hard done by starting their careers during COVID-19, as their learning opportunities, training and team building was shifted due to working from home:

“*… but I do feel I do feel quite robbed of my training. I feel like if COVID hadn't come about I would have been a better lawyer, or better candidate attorney, but that's okay”* (P8, black, Bachelor of Law Degree, working in the legal industry).

However, two participants stated that they a positive experience starting work during the COVID-19 pandemic.

“*Well, it was actually quite, it's going to sound very bad, but it was actually quite nice for me. I didn't need to stand up six o'clock to go into work”* (P2, White, Bachelor of Commerce in Informatics and Economics, working in the Information Technology Industry).

For one participant, there was a sense of gratitude that he still had a job as many people around him had lost their jobs. Two participants demonstrated a mixed feeling about starting work during the COVID-19 pandemic:

“*It's been a mixed bag”* (P1, White, Bachelor of Commerce (Honors) in Marketing Management, working in the FMCG industry).

Five participants reported that the COVID-19 pandemic influenced the connections that they had with their co-workers.

“*So, there's been a lot of change in terms of can I say that connectedness with people and different people connect differently, some it's a big party some it playing soccer, some people it's just having a coffee and getting to know each other. So I think that connectedness has been disrupted”* (P1, White, Bachelor of Commerce (Honors) in Marketing Management, working in the FMCG industry).

Additionally, the connectedness theme can be expanded to include the internet connectivity that was needed for the participants to complete their work. Two participants explained that they missed out on building relationships with their co-workers. Additionally, one participant had missed the in-person interactions that he had with his team outside of work.

Finally, a main theme that is demonstrated through the participants' experience transitioning from university to the workplace during the COVID-19 pandemic is working from home. Moreover, one participant noted that being able to work remotely had shown people and companies that that was something that could be implemented in the future. However, some participants stated that as they were new to the working world and the company, they struggled to get in-person guidance on the running of the company as well as the training and learning that they needed at the beginning of their careers.

For one participant, working from home was something that caused stress and tension within his personal life. He stated that the boundaries of work and home were absent, and due to the nature of his work it was easier for him to work the longer hours (Table 4).

**Table 4:** COVID-19.

**Frequency**	**Code**	**Participant**	**Category**	**Frequency**
6	Negative experience	P4, P7, P8, P9, P10	Experience starting work during the COVID-19 Pandemic	10
2	Positive experience	P2, P3.		
2	Mixed feelings	P1, P6		
5	Connectedness	P1, P4, P5, P6, P10	Connectedness	5
2	Relationship building	P2, P6	Relationship building	2
6	Working from home	P1, P2, P3, P6, P7, P8	Working from home	6

#### 6.1.5. Participant's future career

All of the participants demonstrated some idea regarding what their future career looks like (Table 5). Six participants demonstrated that they have a positive view of their careers:

“*I think in the future I want to be in like a working environment constantly, you know, seeing new projects and new things and stuff”* (P3, Indian, Bachelor of Engineering in Industrial Engineering, working in the Consulting industry).

**Table 5:** Future career.

**Frequency**	**Code**	**Participant**	**Category**	**Frequency**
6	Positive	P2, P3, P4, P5, P9, P10	Positive view of future career	8
2	Impact	P1, P5		
3	Negative	P6, P7, P8	Negative view of future career	3
1	Long term view	P3	Change in career	5
4	Change in career path	P4, P5, P7, P10		

Two participants stated that they wished to make some impact on their communities or on others with their careers. However, three participants had more of a negative view of their future careers. Two of the participants stated that they had not thought that far ahead in terms of what they would like to do, with one stating that he was not one to think about his career:

“*I don't have a career vision, then I think I have a vision for other things in life that I'm doing, but not, not my career.”* (P6, Black, Master of Science Degree in Environmental Science and Remote Sensing, working in the ICT industry).

All but one of the participants stated that they did not see a long future with their current company. Four participants expressed a desire to change their career paths. These participants stated a view to change the industry or sector they work within, but all wished to stay within the same type of work that they studied or currently doing (Table 5).

#### 6.1.5. Participant's mental health

Mental health was a common theme to occur during the data gathering process (Table 6). The participants demonstrated categories or themes surrounding mental health and well-being.

**Table 6:** Mental health.

**Frequency**	**Code**	**Participant**	**Category**	**Frequency**
4	Mental health	P4, P5, P8, P10	Mental health and well-being	13
6	Stress	P3, P4, P7, P8, P9, P10		
1	Burnout	P8		
2	Panic	P9, P10		

Four participants alluded to their overall mental well-being and that they needed to deal with a lot of new pressures associated with the working world and that the working environment also had taken a toll on their mental well-being:

“*I think I'm just a functioning psychopath, at this point in time, I think everyone in my team is. We cannot thrive in that high paced environment, but we are dying, health wise and mental wise because the strain has been too much to handle”* (P4, Black, Bachelor of Engineering in Mechanical Engineering and a Bachelor of Engineering (Honors) in Industrial Engineering and working in the Consulting Industry).

Six participants mentioned that they experienced increased stress during their first year of working. The participants expressed the ways in which they handled the stress, either dealing with the challenge or “blanking” out the stressful moments.

“*… but I think when it comes to dealing with a very difficult challenge or problem my first reaction is to actually stress, (laugh) so I do start, I do start stressing.”* (P3, Indian, Bachelor of Engineering in Industrial Engineering, working in the Consulting industry).

For two participants the COVID-19 pandemic caused their stress levels to rise. Furthermore, one participant described the nature and environment of their work as situated in a stressful industry. Another participant experienced burnout due to the amount of work that they needed to complete and the duties they had to cover of another role. Two participants tended to panic at first when they faced with a new or stressful situation. They however added that once they took a step back and analyzed the problem or situation, they were able to overcome the panic that had initially set in.

### 6.2. Manageability

The manageability theme describes the participants' belief that they have the resources they need to manage stressful life events or demands.

#### 6.2.1. Understanding how participants manage their job

To determine how the participants cope with the demands of their jobs during the transition, they were asked how they managed their jobs and the demands that came with it (Table 7).

**Table 7:** Managing the job.

**Frequency**	**Code**	**Participant**	**Category**	**Frequency**
8	Planning	P1, P2, P3, P6, P7, P8, P9, P10	Planning	8
6	Help	P2, P3, P4, P5, P7, P9	Asking for help	6
4	Working until the work is complete	P4, P5, P6, P8	Work	10
3	Challenge	P4, P5, P6		
3	One day at a time	P5, P8, P10		

Eight participants demonstrated that planning their days was one way of managing their jobs. The planning was centered on creating to-do lists or making use of a calendar system and ensuring the work that needed to be done got done. Furthermore, the planning assisted the participants to determine which tasks were of a priority and needed the most attention.

“*I think it's just prioritizing. You know it's so cliché when people say yeah time management, but that is actually a real thing”* (P9, White, Bachelor of Arts (Law) and Bachelor of Law Degree, working in the legal industry).

Six participants stated that they asked for help when they did not know how to complete a task or when they had challenges in understanding the task. Four participants demonstrated that they managed their jobs by working until the task at hand had been completed. The participants stated that they had hard time leaving work unfinished and felt they needed to complete all assigned work, even if it meant working outside the standard working hours. Three participants enjoyed a challenge in their work. One participant indicated that when confronted by a challenge, he enjoyed tackling and overcoming it.

“*How do I navigate myself through challenge? Umm brute force. Brute force”* (Participant 4, Black, Bachelor of Engineering in Mechanical Engineering and a Bachelor of Engineering (Honors) in Industrial Engineering and working in the Consulting Industry).

However, two participants stated that they enjoyed working through challenges that enabled them to try new things which ultimately helped them learn something new. Three participants stated that they managed their jobs by taking it 1 day at a time. In this way, they stated that they focused on what needed to be completed on a given day.

#### 6.2.2. Organizational support

Support provided by organizations is an important component of how their employees manage and cope with job demands (Table 8). Six participants were provided with positive support during their transition from university to the workplace.

“*So that was one way of support I think also, I was given a lot of opportunities to learn a lot of skills, which I never thought I would need so if it was presentation skills, building the presentation, actually presenting”* (P1, White, Bachelor of Commerce (Honors) in Marketing Management, working in the FMCG industry).

**Table 8:** Support.

**Frequency**	**Code**	**Participant**	**Category**	**Frequency**
5	Support	P1, P5, P6, P4, P10	Support provided by the organization	15
5	Mentor	P1, P2, P3, P6, P10		
3	Induction	P1, P2, P10		
2	Finances	P2, P8		
3	Graduate program	P4, P5, P6	Graduate program	3
5	Team members	P3, P6, P7, P9, P10	Support from team members	5

Participant 6, who joined a graduate program, stated that his organization provided ample support by growing their skills and knowledge. However, participant 4 stated that despite his organization thinking that it supported the graduate employees, he felt that this was not the case and wished for more support.

Five participants emphasized that they felt their organizations supported them by having senior employees as mentors. As such, the participants could turn to them for guidance and learn from them. Furthermore, some participants were also given “buddies” who were also going through the same process and transition as them. Three participants commented that their organizations provided a useful induction process at the beginning of their employment. This induction process allowed the participants to learn about the company. Additionally, for one participant, an induction project was given to him during this process which assisted him in developing his skills. Two participants stated that they would have preferred to join a graduate program for their first employment position. They stated that they felt like they would have benefited more in if they were in a more structured program as it would have exposed them to all they were expected to know in the field that they were in. On the other hand, participant 6 joined a graduate program and stated that is provided him with the support he needed during his transition from university to the workplace. Another two participants highlighted that they appreciated the financial support in the form of competitive salaries that they received from their organization, which helped ease the financial stress that others may face. In addition, one participant stated that the organization financially supported him by paying his law school fees as well as the Board Examinations. Finally, the participants emphasized that they were able to manage this process with the support of their co-workers. The participants revealed that that they could count on their colleagues and senior members if they did not understand their tasks or were having difficulty accomplishing them.

#### 6.2.3. Coping techniques

The coping techniques that the participants use at their disposal is an important component that falls within the manageability theme (Table 9).

**Table 9:** Coping.

**Frequency**	**Code**	**Participant**	**Category**	**Frequency**
5	Drive	P1, P6, P7, P8, P10	Drive	5
4	Learning	P1, P5, P6, P7	Learning	4
1	Recognition	P1	Recognition	1
7	Socializing and drinking	P1, P4, P5, P7, P8, P9, P10	Non-work coping techniques	23
6	Physical activity	P1, P2, P5, P7, P8, P9		
4	Watching series/art shows	P4, P6, P7, P9		
3	Taking a step back	P2, P5, P8		
1	Meditation	P1		
1	Religion/faith	P8		
1	Smoking	P10		

Five participants all stated they had an inner drive to cope with the stress, pressure and tasks that they needed to complete in their positions. The drive expressed by the participants stemmed from their need to succeed, their enjoyment of their jobs and need to learn as much as they could in their first few years of working.

“*I think it's quite a few things I think number one, I think, I want it, I want to succeed, I want to grow, I want to learn, I want to try, so I'd say inner motivation to want to do it”* (P1, White, Bachelor of Commerce (Honors) in Marketing Management, working in the FMCG industry).

The participants all stated that their first 2 or 3 years of their career was involved immense learning opportunities hence their main focus was on learning what they needed for their future. Participant 1 stated that he coped with the demands of the job through his own recognition of what he had achieved and the recognition he received from his seniors for the work that he was doing.

The participants provided their non-work coping mechanisms when faced with the stress Seven participants stated that they coped with the stress by socializing and talking with friends and family. The socializing involved having a drink and alcohol. Additionally, six participants stated that doing physical activity assisted them in clearing their minds after a long day at work and de-stressing. Three participants stated that playing online video games with their friends which helped them to cope with the work stress and take their mind off their tasks. Additionally, four participations stated that watching movies or series assisted them in de-stressing. They stated that by watching series or art shows they were able to clear their minds and not think about work.

Four participants stated that they dealt with their stress by taking a step back to determine what was causing it, before tackling the stress that they were experiencing. They added that they did not like to dwell too long on the stress that they were experiencing and as such, moved on quickly. They also put a divide between work and personal life by for example putting their work computers away. Additionally, one participant stated that he used meditation to clear his mind and de-stress when he was experiencing a lot of stress at work. Another participant coped by turning to his religion and faith. Participant 9 stated that doing something that is not work-related was a way for him to cope. Finally, participant 10 stated that he coped with the stress at work by smoking hookah.

#### 6.2.4. Dealing with change

Throughout the transition period, the participants deal with a lot of changes that are happening in their lives (Table 10). Therefore, the participants stated the ways in which they dealt with the stress and the coping mechanisms that they used. Some participants stated that they took a moment to digest what was happening and think about the situation before acting on it. Other participants stated that they resorted to their coping mechanisms to deal with new changes that were happening while some stated that they enjoyed changes and adapted quickly to them. The participants added that through these changes they are able to learn either new skills or from the situation itself:

“*I like to digest them. I like to really get into it or think about it, or experience it, before I make an opinion. Even though it's, it's rather difficult to sometimes be objective, and that there is change and uncertainty let's wait and see how it goes”* (P1, White, Bachelor of Commerce (Honors) in Marketing Management, working in the FMCG industry).

**Table 10:** Dealing with changes.

**Frequency**	**Code**	**Participant**	**Category**	**Frequency**
1	Digest the changes	P1	Digest the changes	2
1	Focusing on what needs to be done	P6, P9		
1	Learning	P5	Learning	1
1	Support mechanisms	P1	Support mechanisms	5
1	Comedy	P4		
1	Patience	P8		
2	Attitude	P9, P10		
1	Asking for help	P9	Help	1

### 6.3. Meaningfulness

The meaningfulness theme indicates if an individual believes that these life events or demands are worthy and meaningful to engage with and spend time and effort dealing with them (Table 11). Therefore, it concerns the participants' motivation to cope with the stress or the transition that they are experiencing. This also concerns the satisfaction that they are getting through this transition and if the transition from university to the workplace is worthwhile for their future careers.

**Table 11:** Meaningfulness.

**Frequency**	**Code**	**Participant**	**Category**	**Frequency**
2	Identity of the participants	P1, P10	The participants nature	3
1	Curiosity	P6		
1	Gratefulness	P3		
3	Learning	P2, P6, P7	Learning	3
3	Giving back to the community	P5, P9, P10	Giving back to the community	3
3	No meaning	P4, P7, P8	No meaning in their position	3

#### 6.3.1. Meaningfulness at work

The participants explained what they considered as meaningful in their jobs. For two participants, they viewed their job as part of their identity that they were working toward, which made it meaningful to them.

“*… what makes it meaningful is is knowing that I had studied all of these years to be an attorney. And I dreamed of being an attorney. And that's what I'm working toward.”* (P10, Black, Bachelor of Law Degree, working in the legal industry).

Three participants demonstrated that they found meaning in what they were learning and their continuous learning throughout the transition from university to the workplace and in their job. One participant stated that the meaningfulness of his work came from his curiosity. This curiosity was due to his nature in wanting to learn and know how things work. Another participant demonstrated that the meaningfulness stemmed from his gratefulness of having a job in the current South African economic climate. Due to the COVID-19 pandemic, the job losses and the economic climate in South Africa, many of his peers did not have a job; therefore, having a job made it meaningful to him.

Three participants further stated that their work allowed them to give back to their community. The contribution that they felt they were making in their communities made their job meaningful. This contribution, they felt, was both for those in need as well as for the economy of South Africa. However, two participants reported that they did not find their jobs meaningful. They felt that their jobs had not given them too many moments to feel meaningful. They stated they felt that the job they were doing was not for them; as such, they did not find their jobs meaningful:

“*You want to know the truth. I don't find meaning in my job”* (P4, Black, Bachelor of Engineering in Mechanical Engineering and a Bachelor of Engineering (Honors) in Industrial Engineering and working in the Consulting Industry).

Another participant stated that they did not find meaning in their job as it did not have an impact on other people's lives. One participant went to say that he was only staying in the position due to his family's financial situation.

#### 6.3.2. Changes to increase meaning

The study explored the areas in which the participants wished they could change to make their jobs and ultimately their transition period more (Table 12). For two participants they stated that they would enjoy more knowledge or skill sharing with other departments and their co-workers.

**Table 12:** Changes.

**Frequency**	**Code**	**Participant**	**Category**	**Frequency**
2	Knowledge and skills	P1, P4	Collaboration	5
1	Proactive communication	P1		
2	Boundaries	P6, P8		
3	Finances	P2, P5, P8	Finances	3
1	Working from home	P3	Flexibility	1
3	Impact	P5, P8, P9	Giving back to the community	3
1	Transformation	P10	Transformation	1

Financial situation was a common theme among the participants. For three participants they alluded that getting more benefits, or a bigger salary would add more meaning to their jobs and the transition from university to the workplace. They were of the view that receiving better compensation could relieve the financial stress that participants faced.

“*Umm, firstly getting more money”* (P5, Colored, Bachelor of Engineering in Civil Engineering, working in the Consulting Industry).

Due to the COVID-19 pandemic, working from home was an enforced change experienced in the working world. One participant stated that he would find more meaning in his work if he was able to have some days to work from home when back to office policies are reinstated. This level of flexibility would allow the participant to complete their work tasks as well as other tasks outside of work. Three participants stated that they would experience more meaning in their positions if their work was centered on giving back to the community and those in need, particularly to the South African community and people and not to companies. Two participants stated that they would enjoy their work more if their jobs had clearly defined boundaries. These boundaries had to be centered on the participants' job duties and the working hours that the participants were required to work. Finally, one participant mentioned that transformation at top management would add more meaning to his work as this would give him the belief that he could also become a top management lawyer.

## 7. Discussion

### 7.1. How graduates strive to understand their new work environment

The overall findings involving comprehensibility suggest that the graduates described transitioning from university to the workplace as relatively easy or smooth. In addition, the findings illustrate that this change was the most significant adjustment made by them. This sub-theme includes the changes that the graduates need to make in the amount of hours they are required to work and their confidence in their abilities, skills and knowledge to complete their work. It also includes their experience or lack of experience in the workplace and ultimately how they complete the required tasks while faced with the uncertainty of what is expected of them in terms of their job roles. These factors are all considered to be external to the control of the graduates. As such, the graduates feel as though they are unable to cope with the stress associated with the change from university to the workplace. Therefore, the demands that come with their work may cause more stress due to graduates feeling that they do not have the required experience or due to lack of adequate time or ability to complete the tasks, a view shared by Geirdal et al. (2019). Additionally, a handful of graduates demonstrated that they lacked confidence when completing their work. This is as found by Baytiyeh and Naja (2012), who state that a self-confidence challenge factor resulted in graduates finding their transition from university to the workplace as challenging.

The COVID-19 pandemic played a crucial role in the experiences of the graduates transitioning from university to the workplace. Due to the restrictions implemented to help prevent the spread of the COVID-19 virus, participants had to work from home for the first years of their careers. The findings illustrate that most of the graduates struggled with working from home, as they were unable to get assistance in a timely and efficient manner and were working longer hours. The boundaries between work and home were being crossed, which could affect the graduates' mental health resulting in stress. As reported by Grant et al. (2013), over-working remotely could affect employees' health, resulting in the employee burnout. Palumbo (2020) adds that working from home may disrupt the work-life balance of remote employees.

The findings illustrate that the graduates' relationship with their co-workers and managers was affected by working from home, since they could not get assistance in a timely and efficient manner. Additionally, they could not build strong relationships at work due to the minimal face-to-face contact and the absence of relationship-building activities. As a result, the quality of the leadership that the graduates are exposed to, resulting in them not fully understanding what is required from them, was decreased. This finding is supported by Chen and Sriphon (2021) who demonstrated that remote working had disrupted the communal and social exchange relationship between employees and their managers.

The findings further illustrate that most of the graduates had a positive view of their future careers. These findings align with Tuononen et al. (2019) who reported that graduates who work in the field they have studied have increased job satisfaction. Yet although some graduates express that they would like to change their career path in the future, their work would still be centered on what they studied in university. On the other hand, two graduates stated that they would like to change the nature of their work due to them not doing what they had either studied or not enjoying the work that they are doing. This finding is echoed by Tuononen et al. (2019), who state that graduates wanting to change career paths may be due to having difficulty in finding the right opportunities to work in a field related to their studies.

### 7.2. How graduates mange the stress and activate their resources

The findings suggest that the graduates believe that they have the necessary resources to manage the challenges and changes they experience as they transition from university to the workplace. The findings illustrated that the graduates tend to manage their work by either working until the work is just completed, always looking for a way to challenge themselves or taking it 1 day at a time.

The graduates also ask for help from their co-workers or managers if they are unsure how to complete a task. Furthermore, how the graduates plan their days and time also provides a way to manage their jobs. These approaches by the graduates can be considered tools used to manage their jobs, which give them the ability to cope with the stress and the changes as they experience transition from university to the workplace. This finding is supported by Wijk et al. (2020), who suggest that manageability may be created through various tools. Moreover, the findings suggest that the graduates look at their work as a challenge and enjoy being able to challenge their knowledge, skills and capabilities. Therefore, the graduates consider this challenge component to provide them with the motivation to manage their jobs. This was the finding by Johnston et al. (2013), who suggest that individuals can use challenging stressors to experience positive emotions, motivation and productive coping, instead of hindrance stressors.

In addition, the findings demonstrate that the organization's role in supporting the graduates to manage their jobs is one of importance. The findings show that the graduates who felt that their organization supported them effectively managed their work tasks and, ultimately their jobs, during this transition period. The support from the organization can be in the form of an induction process at the beginning of the graduates' jobs, a time-based graduate program, and a buddy system or mentor program with other co-workers. Moreover, some graduates felt that their organizations financially supported them alleviate financial stress, especially during the COVID-19 pandemic, while others felt that they needed more financial support. As stated by Vogt et al. (2016), these findings assert that when employees feel that their work and environment provide them with the necessary social support and opportunity to develop their work, their SOC levels and ultimately their manageability level will allow them to integrate into their work seamlessly. Therefore, the job resources that the graduates have been provided with will help them assimilate into the company's environment and strengthen their manageability levels (Vogt et al., 2016).

Further, the graduates turn to non-work techniques to cope with the stress they experience at work as they transition from university to the workplace. These coping techniques were found to include socializing with friends and family, engaging in physical activities, watching series or movies and taking a step back to re-center themselves. By making use of physical activity exercises, the graduates can effectively deal with their stress outside of work. This is a view shared by Stults-Kolehmainen and Sinha (2013), who suggest that when individuals engage in exercise, they use it as a form to deal with or steer away from the stress they are experiencing. In addition, the findings illustrate that engaging in activities such as meditation, watching series or movies and socializing with friends and family can be an effective way for the graduates to manage their stress. This finding is supported by Kumar and Bhukar (2013) and Kebbi and Al-Hroub (2018), who state that individuals who meditate, turn to their family and friends for support and take days off to relax, tend to manage the stress they experience at work more effectively.

### 7.3. How graduates give their new work situation meaning

The findings illustrate that the graduates view their jobs mainly as meaningful. The findings demonstrate that the graduates find meaning in their jobs due to who they are, how they see themselves and the nature of their minds. More specifically, this suggests that the graduates find meaning in doing their jobs as it forms part of their identity. Thus, it can be said that the graduates are developing or changing their work identity from that of a student to a professional. This finding is supported by Smith et al. (2014) who suggest that students change their self-perceptions, their skills and capabilities and how they work following their graduations, and when they start working. However, the findings also suggest that some graduates did not find their work meaningful. In this case, the findings show that these graduates view their work as just one to get done, and do not find the tasks meaningful. It could be due to different expectations that the graduates had pertaining to the job or due to them not seeing their work contributing to anyone or to the community. As Wijk et al. (2020) found, this view by the graduates have may be due to the different perceptions that they would have had from university to the workplace.

Furthermore, the results illustrate that knowledge and skill-sharing between the graduates and their more experienced co-workers would enable them to develop more meaning in their work. Additionally, the findings also demonstrate that the graduates would like to do more work for the communities in need hence positively impacting or helping others. Furthermore, the theme of transformation in the South African workplace would add more meaning to the graduates. In this sense, the graduates expressed that if they could see a person of color in a management, then they would believe that they could make it into a managerial position too. Hence, having these role models to look up to in the workplace would provide meaning to the graduates.

Finally, the participants expressed that despite experiencing some stress during the transition period, they were able to cope effectively with it. This finding is supported by Oosthuizen and van Lill (2008) and Wijk et al. (2020), who suggest that one could expect that individuals with a relatively strong SOC would cope more effectively with stress than those who have a relatively low SOC.

## 8. Conclusion and recommendations

This study aimed to understand the SOC components in the context of young male graduates transitioning from university into work life during Covid-19. It contributes to understand how male young graduates apply comprehensibility, manageability and meaning to their life situation to deal with stressors in their new work environment in South Africa. The research shows that male graduates understand their transitioning into the workplace and its challenges. They know how to manage it and thereby transform their stress experiences into a manageable process from which they can learn when they use their resources to counteract the challenges. Finally, they show that they apply meaningfulness toward their transition from university into the workplace, are motivated and manage the process well.

To understand the transitional process, graduates primarily reflect upon the transitional process and aim at understanding their new, specific work place as well. They in particular connect to their co-workers within the new work environment to understand the processes and the organization and are open to learn. They are aware of the challenge of COVID-19 and how it impacted on their transitioning, of the challenges of connecting to others and how COVID-19 impacted their relationship building within the new context and their personal mental health and well-being.

Their manageability was mainly impacted through the actual work they had to perform in the new context, their ability to plan and structure their work and their openness to ask for help and support provided by the organization. To manage their work, they predominately relied on non-work coping techniques, that means their personal coping strategies. This is a major finding in this study, because it shows that the manageability in the end is related to their personal coping strategies and not the organizational support as much as it could have been expected.

Finally, meaningfulness played a role in managing stress. Thereby meaningfulness to transition into the work field was related to personal meaningfulness concepts, the idea to give back to the community and the job position-related meaning-making. Meaningfulness in the job transition increased through collaboration and pay.

It can be concluded that comprehensibility and thereby the understanding of the transitional processes were the most important aspects for male graduates to manage stress. This was followed by the ability to manage the transition primarily through the application of personal resources and coping mechanisms. Finally, the ascription of meaningfulness was also important to manage stress, but not as much as the other two components.

The findings support previous research in the field of SOC in organizational contexts. It contributes to reinforcing the idea that the personal stress management and coping is predominately ascribed by young graduates to their own, personal abilities to deal with challenges and stress and not as much to the organization, its processes and structures to integrate graduates into the new workforce. That means, on the one hand, that organizations could reach out more to graduates and present supporting structures—especially to those who do not have the personal strengths and coping mechanisms to deal with change and transitions well on their own. On the other hand, it shows that the higher education system needs to focus on preparing young graduates even more for this transition by training their personal skills and coping mechanisms to keep mentally healthy during challenging times on the job and job transitions.

Finally, more research is needed to compare male graduates and their coping with transitions into the work force across cultures. Thereby, also other intersectional aspects (age, gender, socio-economic background, job and background of parents etc.) should be taken into consideration to explore which intersectional aspects do influence the comprehensibility most in young male (and female) graduates. Finally, research needs to further explore how to increase manageability and meaningfulness in transitional contexts within different cultures and country setting (e.g. developed and developing country-contexts). Based on this future research, trainings for graduates can be developed and conducted even with gender-specific aspects to make the transition into the work force even less challenging and increase graduates' mental health and well-being even more.

## Data availability statement

The raw data supporting the conclusions of this article will be made available by the authors, without undue reservation.

## Ethics statement

The studies involving human participants were reviewed and approved by University of Johannesburg. The patients/participants provided their written informed consent to participate in this study.

## Author contributions

KF collected the data. Both authors contributed to writing up this article. All authors contributed to the article and approved the submitted version.

## Footnotes

^1^Descriptions of participants are as follows, e.g.: Participant 1 is abbreviated as P1.
